# The within-host dynamics of African trypanosome infections

**DOI:** 10.1098/rstb.2014.0288

**Published:** 2015-08-19

**Authors:** Keith R. Matthews, Richard McCulloch, Liam J. Morrison

**Affiliations:** 1 Centre for Immunity, Infection and Evolution, Institute for Immunology and Infection Research, School of Biological Sciences, University of Edinburgh, West Mains Road, Edinburgh EH9 3JT, UK; 2 Wellcome Trust Centre for Molecular Parasitology, Institute of Infection, Immunity and Inflammation, College of Medical, Veterinary and Life Sciences, University of Glasgow, Sir Graeme Davies Building, 120 University Place, Glasgow G12 8TA, UK; 3 Roslin Institute, Royal (Dick) School of Veterinary Studies, University of Edinburgh, Easter Bush, Midlothian EH25 9RG, UK

**Keywords:** *Trypanosoma*, trypanosome, antigenic variation, transmission, quorum sensing, within-host dynamics

## Abstract

African trypanosomes are single-celled protozoan parasites that are capable of long-term survival while living extracellularly in the bloodstream and tissues of mammalian hosts. Prolonged infections are possible because trypanosomes undergo antigenic variation—the expression of a large repertoire of antigenically distinct surface coats, which allows the parasite population to evade antibody-mediated elimination. The mechanisms by which antigen genes become activated influence their order of expression, most likely by influencing the frequency of productive antigen switching, which in turn is likely to contribute to infection chronicity. Superimposed upon antigen switching as a contributor to trypanosome infection dynamics is the density-dependent production of cell-cycle arrested parasite transmission stages, which limit the infection while ensuring parasite spread to new hosts via the bite of blood-feeding tsetse flies. Neither antigen switching nor developmental progression to transmission stages is driven by the host. However, the host can contribute to the infection dynamic through the selection of distinct antigen types, the influence of genetic susceptibility or trypanotolerance and the potential influence of host-dependent effects on parasite virulence, development of transmission stages and pathogenicity. In a zoonotic infection cycle where trypanosomes circulate within a range of host animal populations, and in some cases humans, there is considerable scope for a complex interplay between parasite immune evasion, transmission potential and host factors to govern the profile and outcome of infection.

## 1. Introduction

**Figure 1.**
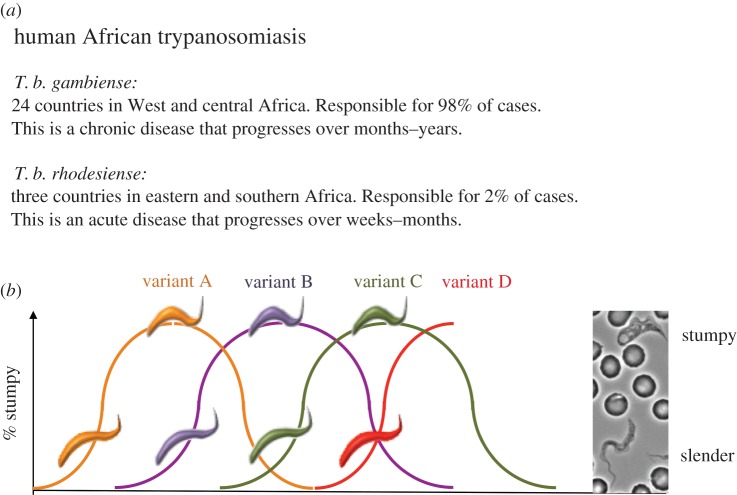
An overview of African trypanosomiaisis. (*a*) Shows a summary of the distribution and disease profile of the two species of African trypanosome responsible for human infection. Animal African trypanosomiasis (caused by *T. brucei*, *T. congolense* and *T. vivax*) is distributed throughout sub-Saharan Africa coincident with the distribution of the disease vector, tsetse flies. (*b*) Shows the conventional view of a trypanosome infection profile. Infection chronicity is achieved by appearance of a progression of waves of parasitaemia with distinct waves being composed of trypanosomes with antigenically distinct coats (for simplicity, each wave is shown as a single VSG, though normally many VSGs are represented per wave). Within each wave of parasitaemia, a developmental switch occurs, whereby proliferative slender forms become arrested stumpy forms as parasite numbers increase in response to the accumulation of the quorum-sensing signal, SIF.

African trypanosomes are protozoan parasites of a range of mammalian hosts, infecting humans, livestock and wild animal reservoirs. Trypanosome infections continue to have important consequences for health and economic prosperity within afflicted regions [1,2] (figure 1*a*). The parasites are transmitted by blood-feeding tsetse flies, where the parasite undergoes development and non-obligatory sexual exchange [3] prior to inoculation into a new mammalian host in the saliva of the infected fly. Unusually for blood-borne protozoan parasites, trypanosomes exist extracellularly throughout their life cycle, meaning that not only must they resist innate immune responses but they must also overcome continual exposure to the humoural immune responses of their mammalian hosts. Thwarting adaptive host immunity allows trypanosomes to survive and establish chronic infections, enhancing transmission and dissemination. They achieve this through an extreme capacity for antigenic variation, allowing the parasite population to evade host antibody responses for months to years (figure 1*b*). The molecular mechanisms of trypanosome antigenic variation have been the subject of intense research for over three decades and are the subject of several recent reviews [4–7]. In recent years, however, the sophistication of the parasite's strategies to achieve infection chronicity, and so increase their capacity for transmission, have become clear, revealing a complex interplay between the parasite's strategies for immune evasion, their developmental control of transmission potential and the contributions of host immunity in a field setting where hosts can be continually exposed to trypanosome infections [8–12]. The interactions between these different parasite and host components of the infection dynamic are likely to drive responses that shape the epidemiology and evolution of the infection over time and in different geographical settings. This review seeks to summarize how the different contributors to the trypanosome infection profile operate and interface to create a complex and finely balanced host–parasite interaction.

## 2. Antigenic variation

In the mammalian bloodstream, the surface of the African trypanosome cell is completely enshrouded by a homogeneous protein coat comprising a single variant surface glycoprotein (VSG) type [13]. The VSG is a glycophosphatidylinositol-anchored glycosylated protein that shields common and invariant antigens on the parasite surface from the immune system [4] and protects the parasite from complement activated by the alternative pathway [14]. Although the key component of the parasites' immune evasion strategy, the VSG is highly immunogenic. Specifically, an antibody response is raised to epitopes on the exposed N-terminal domain of the VSG, resulting in parasite lysis by the classical pathway of complement activation [15]. This, however, does not clear the infection as a proportion of parasites switch to the expression of an antigenically distinct VSG, which is not recognized by antibodies raised to earlier antigen types. Experimentally, at least 100 antigenically distinct coats have been observed to be expressed from a single infecting trypanosome [16], but this is undoubtedly an underestimation due to detection limitations. In reality, the trypanosome's potential for the expression of distinct antigenic types may be almost limitless, due to the possession of a huge archive of VSG genes and highly flexible ‘switching’ mechanisms that allow new VSGs to be activated during antigenic variation.

The expression of a given VSG gene depends upon its location within an active telomeric VSG expression site, of which there are potentially 15–25 in the trypanosome genome [17,18], each with a different VSG. Only one expression site is fully active at a time [19], this being uniquely associated with a sub-nuclear transcription factory, the expression site body [20,21]. In addition, a complex interplay between epigenetic silencing factors [22], telomere factors and nuclear envelope association act to ensure allelic exclusion and inactivity of the other expression sites [5]. Active expression sites are transcribed by RNA polymerase I [23] and several expression site-associated genes (ESAGs) are co-expressed with the VSG gene in the same polycistronic transcription unit [24–26]. The multiplicity of VSG expression sites means that expression of a new VSG gene can occur through a transcriptional switch that activates a new expression site and silences the previously active site. However, by far the most common route of VSG coat switching involves recombination (approx. 90% of switching events [27]), mainly through gene conversion events in which a silent VSG gene is copied and replaces the expressed VSG in the expression site. It is this type of VSG switching that allows prolonged infections and generates VSG diversity beyond the number of VSG genes in the genome archive.

The scale of the archive of VSG genes in trypanosomes is huge, dwarfing the number of antigenically variant genes in the genomes of other organisms, such as *Plasmodium*, that also rely on antigenic variation for survival. The VSG repertoire has been characterized in two strains of *Trypanosoma brucei* [28–30], where antigenic variation is best described, as well as in the animal infective trypanosomes *Trypanosoma vivax* and *Trypanosoma congolense* [31]. Even though the VSG cataloguing is still incomplete, the *T. brucei* genome can contain more than 2000 VSG genes (more than 20% of the coding genome), of which the majority exist in transcriptionally silent subtelomeric arrays, although a substantial fraction are found in aneuploid minichromosomes. The VSG repertoire appears highly dynamic, with changes in VSG numbers and identities detectable during strain propagation [30], and larger scale rearrangements leading to chromosome size variation within and between strains [32]. Most VSGs are single copy in the archive, sharing little primary sequence homology with other VSGs. However, only approximately 5–10% of the subtelomeric array VSG repertoire encodes intact and functional VSGs, with the remainder either containing frameshifts or truncations rendering them incapable of generating intact VSGs (approx. 80–85%) or encoding predicted features that are atypical of VSGs (10%) [33]. Importantly, the VSG pseudogenes are not non-functional, but make a major contribution to antigenic variation (see below). Nonetheless, the detection of such a large pseudogenic repertoire was unexpected, particularly as other organisms (e.g. *Anaplasma marginale* and *Borrelia burgdorferi*) that use similar gene conversion strategies based on antigen pseudogenes can generate antigenic variation and chronic infections with much smaller gene repertoires (tens of genes) [7]. Moreover, until genome sequencing, the activation of new VSGs based on pseudogenes, though detected [34,35], was thought to be a rare event during trypanosome infections; in fact, it appears to be the major driver of long-term infections and transmission [36–38].

**Figure 2.**
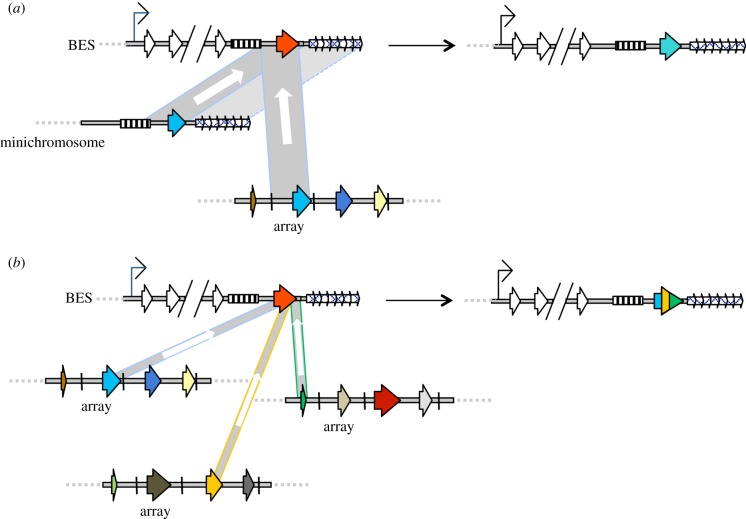
VSG gene conversion during antigenic variation. (*a*) Gene conversion of intact VSG genes into the active bloodstream VSG expression site (BES), using donor VSGs that are either present in a minichromosome or a subtelomeric VSG array. In all cases, the VSGs are shown as coloured arrows and the extent of sequence copied during gene conversion is indicated by a grey box, within which the direction of copying is shown by a white arrow. During gene conversion from a silent minichromosome or array VSG (both light blue), the upstream boundary is normally 70 bp repeats (hatched box), which are found adjacent to most VSGs (though their numbers are lower when associated with array VSGs). The downstream boundary of gene conversion is frequently the 3′ end or 3′ flank of the VSG, though this can extend further when minichromosome VSGs act as donors, including reactions that encompass the telomere repeats (small, arrayed arrows). In the BES, ESAG genes (white arrows) are co-transcribed with the VSG (red) from a common promoter (thin black arrow); silent BES (not shown) can also act as donors of new VSGs by gene conversion, or can elicit VSG coat changes by transcriptional switching (not shown; see text). (*b*) Segmental gene conversion to form novel mosaic VSGs. In this reaction, multiple silent VSGs (here, three: light blue, orange and green) are recombined together to form a new VSG that is a composite of the three gene sequences. The VSGs that act as donors in segmental gene conversion are frequently pseudogenes and are normally located in disparate regions of the subtelomeric array archive. Many details of this reaction are uncertain, and some assumptions or simplifications are made: segmental gene conversion to form VSG mosaics may not happen in the active BES, as shown here; gene conversion is shown to encompass only VSG ORF-internal sequences, but the reactions may be ‘anchored’ by upstream or downstream homology; VSG mosaics normally display much greater intermingling of the donor VSG sequences than is indicated here.

Two different gene conversion reactions contribute to VSG switching (figure 2). In one reaction, an intact, previously silent VSG replaces the complete VSG in the active expression site (figure 2*a*). This reaction is dependent upon flanking sequence homology, perhaps most notably upstream 70 bp repeats that are uniquely associated with approximately 90% of VSGs [37]. The 70 bp repeats delimit the boundary of VSG gene conversion of intact VSGs from the subtelomeric arrays and the minichromosomes. VSGs in the silent expression sites also act as substrates in intact VSG gene conversion and, despite the greater available homology, the increased numbers of 70 bp repeats in these sites mean they are also frequently, though not exclusively, the upstream boundary here [39–41]. The second reaction, termed segmental gene conversion, involves recombination driven by the coding sequence of the VSGs, so enabling utilization of the VSG pseudogenes; here, multiple VSG ORFs (intact, pseudogene or gene fragments) can be recombined (figure 2*b*), creating novel antigens (mosaic VSGs) and therefore multiplying the potential expressed VSG diversity well beyond the limitations of the existing genome's VSG repertoire [37,38]. We have much to learn about this reaction; for instance, unlike VSG conversion of intact genes, which genetic evidence shows is linked to homologous recombination, no factors (*cis*- or *trans*-acting) that act in segmental VSG gene conversion have been described; also, we do not know where VSG assembly occurs in the genome.

The different mechanisms of VSG gene activation (expression site activation, gene conversion driven by flanking repeat sequences, assembly of mosaic genes) have different probabilities that shape the profile of expressed VSGs during a chronic infection [42]. Early in infection, switching between intact telomeric VSG genes seems to predominate. In part, this may be due to transcriptional switching, perhaps as a means to establish active transcription of the most host-appropriate expression site [43]. However, it is also a result of early telomeric VSG recombination, which may reflect the observation that the proportion of such VSGs that are intact and functional, both in the expression sites [18] and in the minichromosomes [30], is much higher than in the subtelomeric arrays. Equally, telomeric location may promote recombinogenic interactions [44]. Activation of intact silent subtelomeric VSG genes by gene conversion using homologous flanking sequences is seen next; because these events are independent of the sequence of the previously expressed VSG, the order of VSG activation is unpredictable. Thereafter, mosaic VSG assembly by segmental gene conversion predominates and sustains the appearance of new antigen types [37,38], this being necessary to maintain the infection in a context where parasites expressing or re-expressing frequently activated VSG are eliminated by antibodies generated during exposure earlier in the infection. In this phase of the infection, recombination within the coding region of VSG genes appears to generate dependency on the previously expressed VSG gene, based on a requirement for relatively closely related VSGs, creating the potential for directionality in the order of activation of VSG genes. Although this has the potential to create a further hierarchy in the appearance of expressed antigens, the complexity of the recombination events and the selection of antigenically novel VSG assemblies by the immune system makes the expressed VSG unpredictable and the available evidence suggests increasing VSG diversity with time [38]; indeed, it is probable that the capacity for such diversity has been significantly underestimated by the experimental approaches used to date. Moreover, as the assembly of VSG mosaics initiates and progresses independently in each infection, different hosts will be exposed to completely different antigen repertoires that would prevent any possibility of generating meaningful immunity to the infecting parasite population or to subsequent co-infecting trypanosome populations.

## 3. Developmental contributions to the infection dynamic

Like many pathogens, the trypanosome must balance virulence (defined here as proliferation in the host), pathogenicity (defined here as damage to the host) and transmission (i.e. the capacity for its successful uptake and establishment in the tsetse fly) to maximize its long-term survival and spread. The trypanosome achieves this by regulating its growth within mammalian hosts in a density-dependent manner [45–47] and through the generation of a specialized developmental stage, the stumpy form, optimized for transmission to tsetse flies [48]. These adaptations are linked because the stumpy forms are non-proliferative and are generated from proliferative slender forms through a quorum-sensing-like process, whereby a soluble parasite-derived factor triggers the developmental transition [45]. The identity of the factor, called stumpy induction factor (SIF) (though it may in fact represent a mixture of factors), is unknown but evidence suggests that it is small (less than 500 Da), heat stable and generated by slender forms, such that it accumulates to provide a measure of parasite density [45]. While the factor is uncharacterized, a recent genome-wide screen identified molecular components of the cellular response pathway leading to the production of stumpy forms [49]. Several protein kinases and phosphatases were revealed as signal transduction components, as well as gene expression regulators (namely predicted RNA-binding proteins) and hypothetical proteins of unknown function. The screen identified drivers of stumpy formation whose genetic depletion or ablation prevented the developmental response regardless of parasite density, complementing earlier studies that had identified molecules able to inhibit stumpy formation [50–52]. Analysis of the identified pathway components has shown similarity to nutrient sensing and developmental responses in yeasts and *Dictyostelium* [49,53], suggesting that the signalling pathways regulating trypanosome stumpy formation share evolutionary origins with fundamental environmental sensing pathways conserved in diverse eukaryotes [53].

The balance between slender and stumpy forms within each wave of parasitaemia and during the course of a chronic trypanosome infection plays a significant part in controlling the infection dynamic of the parasite [8]. Early in infection, the parasites are predominantly slender and numbers rapidly increase [9]. However, the accumulation of SIF drives cell-cycle arrest and then morphological development to stumpy forms, this being accompanied by the expression of several stumpy form characteristics, including the expression of the PAD1 surface transporter that detects transmission of the parasites taken up in a tsetse fly blood meal [54]. Stumpy forms are also somewhat more robust than slender forms: they are more tolerant of pH stress and proteolytic attack [55] (aiding their survival in the tsetse midgut) and are better able to survive at equivalent antibody titres [56,57], this being assisted by their capacity for the clearance of antibody from their surface by hydrodynamic flow [57].

Nonetheless, with sufficient antibody titres, both slender and stumpy forms are destroyed such that the parasite numbers rapidly decline. Despite its stability *in vitro*, SIF must rapidly turn over *in vivo*, allowing parasites that have undergone an antigenic switch to begin to accumulate as proliferating slender forms and thereby re-establish the parasitaemia. In textbook descriptions, this balance between proliferation as slender forms, differentiation to stumpy forms, the immune clearance of slender and stumpy forms and the recrudescence of antigenically distinct slender forms generates regularly periodic waves of parasitaemia. However, a quantitative analysis of parasite numbers and the proportion of stumpy forms in chronic mouse infections demonstrated a more complex infection profile, where distinct cyclical waves of parasitaemia were not obvious later in infection and stumpy forms stably predominated in the overall parasite population [9]. This infection profile has been interpreted to help the trypanosomes to get an early foothold in a mammalian infection, aided by the proliferation and rapid antigen switching of the initial slender population, with the later stumpy-enriched population in a chronic infection prolonging host survival and enhancing the probability of transmission [8]. This model, however, has not been tested in livestock infections where parasitaemias are usually lower than in mice, and in which the textbook infection kinetics of distinct peaks of parasitaemia are more readily identified.

## 4. Balancing virulence and transmission in trypanosome infections

**Figure 3.**
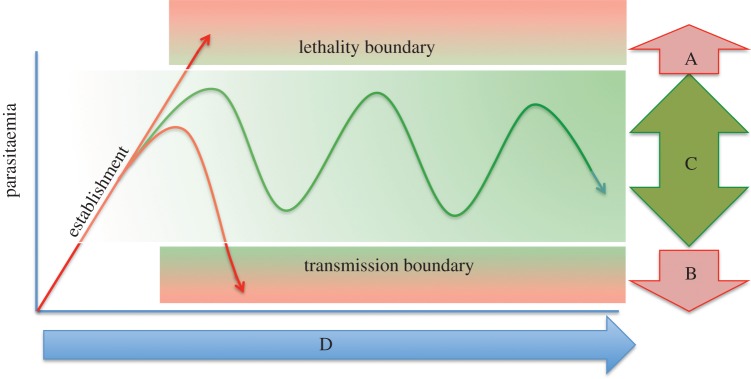
The classical description of the interplay between antigenic variation and infection chronicity (redrawn from [58]). In scenario A, the parasites overgrow and kill the host. Scenario B occurs when parasites are rapidly cleared from the host. Scenario C is characteristic of trypanosome infections and is dependent upon both antigenic variation to evade specific immune responses (prevents scenario B) and density-dependent differentiation of slender to stumpy parasites (prevents scenario A). These processes are parasite-driven and independent of the host. Scenario C maximizes transmission, which will ultimately be the primary selective force on the trypanosome population. Several factors will determine the kinetics of infection in scenario C (i.e. infection duration and total parasite load; D) and these will include host susceptibility, parasite virulence and population factors such as herd immunity and co-infections, and the interplay of these factors with parasite antigenic variation and differentiation. These selective factors will shape the usage and evolution of the VSG repertoire at the individual and population levels.

The development of stumpy forms might serve several beneficial purposes for trypanosomes (figure 3). Firstly, the production of arrested stumpy forms as the parasitaemia accumulates prevents an uncontrolled proliferation of slender forms that would rapidly kill the host. Secondly, the more robust nature of the stumpy form when compared with the slender form would promote its survival upon transmission to the tsetse fly, where the parasite is attacked by the proteolytic environment of the insect midgut. Finally, by reducing the overall proportion of proliferative forms in the mammalian parasitaemia in chronic infections, the overall frequency of antigenic variation would be reduced [9] (as only proliferative forms would undergo productive antigen switches), potentially prolonging the functional within-infection lifetime of the antigen repertoire [9], and this limitation on repertoire usage may also play a role in restricting herd immunity and reinfection at the host population level [8]. Although each of these potential benefits would apparently favour parasite survival and spread, direct evidence for each is limited. For example, while the laboratory selection of parasite lines that can no longer generate stumpy forms does generate virulent lines that rapidly kill the host [59,60], stumpy forms are not detected in other African trypanosome species outwith the *T. brucei* group (e.g. *T. congolense* and *T. vivax*) [61,62]. These species nonetheless are sustained successfully in sub-Saharan Africa and transmitted by tsetse flies, with *T. congolense* entering the fly midgut initially to establish infection. Furthermore, there is little experimental evidence that the proportion of stumpy forms in an infection dominates the likelihood of transmission. While studies clearly support the importance of stumpy forms in transmission [63], the advantage of having 90% as opposed to 10% stumpy forms in a tsetse blood meal is less clear assuming sufficient parasites are ingested. Finally, the significance of restricting exposure to the immune system of different antigen types is unknown. While the rapid expression of many antigen types might unnecessarily expose the parasite's VSG repertoire and so limit infection chronicity, the simultaneous expression of many diverse antigen types might also restrict the production of an effective immune response against any one antigen type [10,64]. This might allow minor types to sustain expression or to be re-expressed later in infection when dominant antigen types are eliminated. Furthermore, the simultaneous expression of many different antigen types in the infection could act to perturb immune efficacy or promote immunosuppression. Following the recent ability unambiguously to identify stumpy forms in an infection using PAD1 as a molecular marker [9], the capacity to identify expressed antigen genes at the population level by deep sequence expression analysis and the tools to activate or inhibit stumpy formation via RNAi or gene overexpression [49], the contributions to each of the different components of the trypanosome infection dynamic are now accessible to experimentation. This places us in an excellent position to understand how trypanosomes contribute to their parasitaemia and the consequences of perturbing different components in the context of acute and chronic infections.

## 5. Contributions from the host

Unusually in pathogen biology, trypanosome research has placed relatively little emphasis on the analysis of host contributions to the context of infection. Largely, this has been because antigenic variation and development in the host are parasite-intrinsic factors. Nonetheless, while the humoral response is clearly necessary to control each variant antigen-expressing subpopulation, host contributions to parasite virulence and the infection dynamic can be complex and host species dependent, limiting experimental tractability outwith the mouse model of infection. For example, populations of livestock and game animals continually exposed to trypanosome infections over time may have the capacity to develop herd immunity, restricting the ability of the circulating parasite population to be transmitted to, and establish in, new hosts [33]. Here, the development of unique antigenic lineages derived from sequential mosaic formation over the course of a chronic infection might play a key role in enabling the parasite to establish in previously or currently infected hosts [12,65]. Similarly, the rapid antigen switching characteristic of field strains of parasites [66] (as occurs early in infections enriched in slender forms) might allow the parasite to probe the immune status of the host until a sufficiently novel antigen type is expressed, allowing the parasite to establish [8]. The diversity and flexibility of the metacyclic VSG repertoire (the VSG expressed from a specialized subset of expression sites activated in the tsetse fly salivary gland in the life cycle stage pre-adapted for mammalian host infection) will also provide increased capacity to infect previously exposed hosts exhibiting herd immunity [67,68]. The metacyclic VSG repertoire is somewhat isolated from the VSG repertoire used in established bloodstream infections, with M-VSG expression sites being shorter, generally lacking in ESAGs and with few or no flanking repeats, limiting their recombination with bloodstream expression sites. Although of more limited diversity (there are around 12–20 M-VSG types [68,69]), the short-term expression of these may limit their immune stimulation, helping the parasite to establish and proliferate until it can switch to expression of antigens from the much larger bloodstream repertoire.

Beyond immunity, there is evidence that the control of trypanosome infections has a strong host component. The most obvious example of this interaction is the inability of most African trypanosomes (including *Trypanosoma brucei brucei*, *T. congolense* and *T. vivax*) to infect human and primate hosts through the presence and expression in humans and primates of trypanolytic serum factors, TLF1 and TLF2, each containing the lytic ApoL1 component of high-density lipoprotein [70]. *Trypanosoma brucei rhodesiense* and *Trypanosoma brucei gambiense* have both evolved independent mechanisms to evade killing in human serum through their expression of SRA [71] and TgsGP1 [72,73], respectively, with a downregulation of the haptoglobin–haemoglobin receptor also contributing to *T. b. gambiense* resistance. Counter selection in some human populations for mutations in ApoL1 generating a form able to kill *T. b. rhodesiense* provides evidence of the evolutionary conflict between humans and trypanosomes, with the trade-off in this case being an increased risk of kidney disease [70,74].

Aside from the binary trait of human infectivity, there is evidence for a genetic basis underlying factors that determine the kinetics and duration of infection, in both parasite and host populations. In mammalian hosts, there is a spectrum of inherent susceptibility to trypanosome infections, ranging from fully susceptible hosts that succumb and die after a short infection timecourse to those defined as ‘trypanotolerant’, or hosts that remain infected but do not develop the severe clinical signs of their susceptible counterparts. At a population level, the variation in these host factors will have a clear influence on parasite transmission, as well as the usage and evolution of VSG repertoires. Animal models of the trypanotolerance phenotype have long been recognized and are particularly well defined in mice and cattle with respect to *T. congolense* infections, but the variation in infection outcome phenotype has also recently been identified in human patients infected with *T. b. gambiense* [75,76], with an infection status analogous to trypanotolerance being characterized [77]. Indeed, genes, alleles and pathways have been identified that are suggested to contribute to host susceptibility in mice, cattle and humans [75,76,78], although it is clear that the phenotype is a quantitative trait in all species with contributions from multiple genes, many of which remain to be identified.

The influence of parasite genotype on infection severity and outcome is also profound, with some strains generating acute and severe infections and others chronic infections with mild symptoms—the classic example being infections in humans with *T. b. gambiense* tending to be more chronic, whereas *T. b. rhodesiense* infections are often very acute. Phenotypes relating to infection severity have been shown to be heritable in the *T. brucei* model in mice, and similar to trypanotolerance in the mammalian host these are quantitative traits with multiple genes involved [79,80]. Therefore, it is clear that the interplay between genes and pathways involved in host susceptibility and trypanosome virulence will have a significant impact upon the kinetics of infection, including parasitaemia, infection profile and infection duration, all of which will interact with antigenic variation and differentiation to shape the within-host dynamics of infection and onwards transmission.

## 6. The impact of co-infections and zoonosis on trypanosome infection dynamics

**Figure 4.**
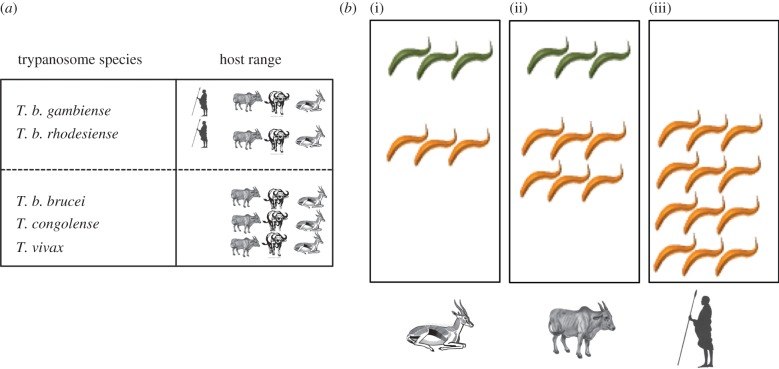
Possible conflicts driving parasite virulence in different host settings. (*a*) The host range of different trypanosome species is shown, with *T. b gambiense* and *T. b. rhodesiense* being human infective. *Trypanosoma brucei gambiense* can also infect livestock, though human infection is more frequently detected; *T. b. rhodesiense* is most frequently found in livestock and game animals. *Trypanosoma brucei brucei*, *T. congolense* and *T. vivax* cannot infect humans but are maintained in game animals and livestock. (*b*) Three scenarios where parasites are transmitted either to trypanotolerant or susceptible hosts, or humans. In each scenario, potential outcomes are: (i) in trypanotolerant hosts, host suppression selects for increased virulence of the parasite population; (ii) parasites might exhibit increased virulence once released from either host suppression in susceptible animals or competition from co-infecting strains; and (iii) in humans, *T. b. rhodesiense* or *T. b. gambiense* are released from inter-species competition and may exhibit increased virulence.

African trypanosomes have the capacity to infect a wide range of mammalian hosts (figure 4*a*). While *T. b. rhodesiense* and *T. b. gambiense* can infect humans, these species also have animal reservoirs, including livestock and game animals, that can probably sustain the parasites in an infection cycle long-term without human involvement. Similarly, with a relatively high frequency of trypanosome infection in animal reservoirs combined with the typically chronic nature of trypanosome infections, the probability of mixed infections between different genotypes or species is significant [81]. This likely generates the potential for evolutionary conflict, particularly in the context of an operating quorum-sensing system. Thus, if two different parasite genotypes exchange density sensing signals, there is the theoretical possibility for selection to occur, whereby the parasites either exploit or perturb each other's signals for their own advantage. This has the potential to select parasites less able to respond to a density sensing signal such that they come to dominate in a mixed infection, or, hypothetically, to even produce a SIF mimic which preferentially limits the proliferation of a competing strain without affecting the producer strain. This conflict could operate to increase the virulence of parasites when competing in a pool of hosts where the parasites are circulating so that they sustain their numbers and maintain their probability of transmission. If removed from the competitive environment—for example, by infecting a new uninfected host, transitioning from a trypanotolerant to a more susceptible host or moving from an animal host to a human host (in the case of *T. b. gambiense* or *T. b. rhodesiense*)—the consequence could be the appearance of highly virulent forms that are more pathogenic to the host (figure 4*b*). The associated trade-off is, potentially, reduced capacity for transmission through their reduced production of stumpy forms in that host [8], but the pivot point when increased virulence and decreased transmission and *vice versa* become strongly deleterious is unknown. Nonetheless, a conflict between virulence and transmission is inevitable either with single or mixed infections, and variable between different hosts, and there is considerable scope for the evolution of systems that provide misinformation to competitors. The extent of the importance or stability of such selection pressures is unclear, but may emerge as the molecular pathways controlling stumpy formation become better understood or the molecular identity of SIF becomes known. This fascinating area is currently unexplored, but the precedent from bacterial intercellular communication systems provides evidence that nuanced communication between different parasites circulating in the same host populations over considerable periods of time will generate novel insights into the factors that control and optimize the infection dynamic of trypanosomes in different mammalian hosts.

## 7. Therapeutic implications

Current drugs licensed to treat trypanosomiasis in humans and livestock are old, have dangerous possible side effects, and resistance is an increasing problem [82]. Consequently, many initiatives to develop new drugs for trypanosomiasis are underway. Most focus on killing the proliferative slender form of the parasite in the bloodstream, but there is also the prospect of targeting parasite development and transmission [83]. For example, depletion of the TOR4 protein in trypanosomes drives the parasites to generate stumpy-like forms in an irreversible arrest [52]. If induced pharmacologically, this would leave affected parasites subject to clearance by the immune system, as would the targeting of other kinases operating on the same pathway [84]. Similarly, molecules that drive stumpy formation could be activated pharmacologically to achieve the same outcome, eliminating the parasite population from the bloodstream [83,84]. In an extension of this approach, inducing parasites to differentiate from bloodstream forms to tsetse midgut procyclic forms in the host bloodstream would render the parasites susceptible to rapid killing by the alternative pathway of complement, as VSG loss is an early component of the differentiation response in the tsetse midgut. An example of this would be targeting the tyrosine phosphatase *Tb*PTP1 whose pharmacological inactivation has been shown to initiate the differentiation of stumpy forms to procyclic forms in the absence of any other external trigger [85]. Likewise, a protein kinase with the same phenotype when ablated by RNAi has also been identified [86]. An important caveat of this approach is that the pharmacological effect must be 100% efficient; otherwise, there will be the potential for the selection of parasites less able to arrest as stumpy forms and so with greater potential virulence [83]. Nonetheless, as reduced stumpy formation could also reduce transmissibility of any resistant parasites, this approach might provide an interesting evolution-resistant therapeutic approach that could provide a useful complement or adjunct to newly developed trypanocidal therapies.

## 8. Perspectives

The capacity of trypanosomes to undergo antigenic variation and development to specialized transmission stages has long been recognized. However, while the molecular mechanisms underlying antigenic variation have been studied in detail over many years, only recently have events been analysed in parasite lines that exhibit full developmental competence. This represents an important step forward, as understanding the interplay between antigenic variation and density-dependent growth control in the mammalian bloodstream is necessary for the dynamics of chronic trypanosome infections to be recognized. These processes are likely to reveal a complex and detailed picture of how the trypanosome sustains itself long-term in mammalian hosts and ensures transmission. Moreover, the interplay between these components has potentially important evolutionary implications likely to have impact on the virulence, transmissibility and epidemiology of trypanosome infections in the field. With the analysis of developmentally competent lines, the identification of molecular components necessary for the control of antigenic variation and developmental progression in the bloodstream, and accessibility of a wider range of field parasite strains (and genome sequences), new insight is likely to emerge rapidly. These insights can be exploited therapeutically or the insight used to understand how therapeutic interventions might fail, or resistance develop. Importantly, the general principles uncovered are also likely to be broadly applicable among a range of related and unrelated pathogens. The interplay between virulence and transmission is complex but fundamental in pathogen biology. Trypanosomes provide one of the most tractable models to understand how this interaction operates, and the implications of perturbing different components on the longevity and spread of the parasite in the field.
